# Reduced Mortality Associated With the Use of Metformin Among Patients With Autoimmune Diseases

**DOI:** 10.3389/fendo.2021.641635

**Published:** 2021-04-23

**Authors:** Chun-Yu Lin, Chun-Hsin Wu, Chung-Yuan Hsu, Tien-Hsing Chen, Ming-Shyan Lin, Yu-Sheng Lin, Yu-Jih Su

**Affiliations:** ^1^ Division of Rheumatology, Department of Internal Medicine, National Cheng Kung University Hospital, College of Medicine, National Cheng Kung University, Tainan, Taiwan; ^2^ Division of Rheumatology, Allergy, and Immunology, Department of Internal Medicine, Chang Gung Memorial Hospital, Kaohsiung, Taiwan; ^3^ College of Medicine, Chang Gung University, Taoyuan, Taiwan; ^4^ Division of Cardiology, Department of Internal Medicine, Chang Gung Memorial Hospital, Keelung, Taiwan; ^5^ Division of Cardiology, Chang Gung Memorial Hospital, Yunlin, Taiwan; ^6^ Division of Cardiology, Chang Gung Memorial Hospital, Chiayi, Taiwan; ^7^ Center for Mitochondrial Research and Medicine, Chang Gung Memorial Hospital, Kaohsiung, Taiwan

**Keywords:** autoimmune diseases, inflammatory disorders, hospital admissions, metformin, mortality

## Abstract

**Objective:**

Metformin has been linked to anti-proliferative and anti-inflammatory mechanisms. In this study, we aimed to examine the long-term impact of metformin on mortality and organ damage in patients with autoimmune diseases and type 2 diabetes mellitus (T2DM).

**Methods:**

We conducted a cohort study using the National Health Insurance Research Database in Taiwan between 1997 and 2013. Based on metformin and other anti-diabetic agent prescriptions, we categorized all patients with autoimmune diseases into either the metformin group (metformin administration for at least 28 days) or the non-metformin group. The primary outcomes were all-cause mortality and annual admission rate, while the secondary outcome was target organ damage. We followed patients from the index date to the date on which the event of interest occurred, death, or the end of this study.

**Results:**

Our cohort study included 3,359 subjects for analysis. During a mean follow up of 5.2 ± 3.8 years, the event rate of all-cause mortality was 228 (33.6%) in the metformin group and 125 (36.9%) in the non-metformin group. The risk of both all-cause mortality and annual number of admissions for autoimmune diseases was significantly lower in the metformin group than in the non-metformin group [hazard ratio (HR) 0.77; 95% CI 0.62–0.96 and risk ratio (RR) 0.81; 95% CI 0.73–0.90, respectively].

**Conclusion:**

Metformin may add benefits beyond T2DM control with regard to reducing all-cause mortality and admission rate, as well as minimizing end-organ injury in lungs and kidneys among patients with autoimmune diseases.

## Introduction

Immunosuppressive agents and corticosteroids have long been a cornerstone of treatment for various autoimmune diseases (AD). However, infectious complications resulting from immunosuppression may result in a high burden of morbidity in patients with AD. A recent clinical trial has demonstrated that the use of metformin in patients with systemic lupus erythematosus (SLE) resulted in decreased disease activity (1). Data regarding whether metformin can be used as a disease modifying treatment in other ADs are scarce (2).

Type 2 diabetes mellitus (T2DM), combined with a number of risk factors of metabolic origin, can result in an increased risk for early mortality (3). Considerable evidence has demonstrated that chronic low-grade inflammation caused by activation of the innate immune system is vital in the pathogenesis of T2DM and major complications, as well as that the antidiabetic drug metformin exhibits various anti-proliferative and anti-inflammatory mechanisms (3). Furthermore, increasing studies have indicated that metformin can reduce cardiovascular mortality (4), cancer mortality (5), all-cause mortality (6), and intensive care unit mortality (7). However, previous studies have rarely investigated the role of metformin in patients with AD and T2DM with regard to mortality (8).

According to the international treatment guidelines for T2DM, metformin is the first-line therapy, followed by dipeptidyl peptidase-4 (DPP-4) inhibitors and glucagon-like peptide 1 (9). Therefore, using a nationwide database, we conducted an observational pharmacoepidemiological study to examine the impact of metformin on mortality and AD-related complications among patients with both AD and T2DM.

## Material and Methods

### Data Source

We obtained real-world data from Taiwan’s National Health Insurance Research Database (NHIRD). NHIRD is an administrative database from Taiwan’s National Health Insurance (NHI) program, which is a compulsory single-payer national health insurance system. The NHI program covers almost all outpatient visits, emergency room services, admission services, medical care services, and prescription drugs. This study was approved by the Institutional Review Board of National Cheng Kung University Hospital (A-EX-109-017) and Chang Gung Medical Foundation (IRB No.: 201600763B1).

### Study Design and Participants

Between 1st January 1997 and 31st December 2013, patients with AD, including systemic lupus erythematosus, rheumatoid arthritis, primary Sjogren’s syndrome, systemic sclerosis, polymyositis, dermatomyositis, anti-neutrophil cytoplasmic antibodies-associated vasculitis, Buerger’s disease, Takayasu arteritis, Kawasaki disease, Behçet’s syndrome, pemphigus, Crohn’s disease, ulcerative colitis, hypersensitivity angiitis, and seronegative spondylopathy, were identified by the corresponding international classification of diseases, ninth revision, clinical modification (ICD-9-CM), and the list of ICD-9 coding is presented in the supplementary table. Most AD diagnoses were confirmed by catastrophic illness certification (CIC) according to NHI program regulations (10, 11). The application of CIC for major AD required a strict review process by two rheumatologists: one application rheumatologist and one anonymous senior rheumatologist as an adjudicator.

T2DM was identified using a physician’s diagnosis with a disease code (ICD-9 CM code: 250) combined with the prescription of glucose-lowering drugs. The accuracy of T2DM diagnosis has been validated in previous studies (12). Only patients that had been diagnosed with AD prior to the first diagnosis of T2DM were included in this study. Patients who had been diagnosed with T2DM prior to the first diagnosis of AD were excluded from this study since metformin may affect the occurrence of AD and become a confounding factor. We further excluded patients with any history of respiratory failure or end-stage renal disease. Study participants were categorized into the metformin group if they had received metformin treatment for at least 28 days. The date of the first metformin prescription was assigned as the index date in the metformin group, while the index date of the non-metformin group was the date of the first prescription of other oral antihyperglycemic agents such as sulfonylureas, α-glucosidase, thiazolidinediones, meglitinides, DPP-4 inhibitor, and insulin.

### Identification of Covariates and Outcomes

Since the NHIRD does not include laboratory test results (i.e., glycohemoglobin, C-reactive protein), we selected several clinical indicators to represent surrogate markers of disease severity, including history of plasmapheresis or plasma exchange, prior admission due to AD, diabetic ketoacidosis (DKA) or hyperosmolar hyperglycemic state (HHS), DM disease duration, and autoimmune disease duration. Such baseline comorbidities as hypertension, dyslipidemia, gout, chronic kidney disease, coronary artery disease, interstitial lung disease, malignancy, stroke, and hepatitis B virus (HBV)/hepatitis C virus (HCV) infections were identified in the period of 365 days prior to the index date by using corresponding ICD-9-CM diagnosis codes in the ambulatory record at least twice or in the inpatient record at least once. We also recorded exposure to medications, including aspirin, clopidogrel, statins, fibrates, anticoagulants, systemic non-steroidal anti-inflammatory drugs (NSAIDs), immunosuppressants (hydroxychloroquine, steroids, cyclosporine, cyclophosphamide, mycophenolate, tacrolimus, azathioprine, methotrexate, sulfasalazine), and antihyperglycemic agents (sulfonylureas, α-glucosidase, thiazolidinediones, meglitinides, DPP-4 inhibitors, insulin), within three months of the index date. Patients were designated as users of certain drugs if they refilled the prescription for the 80% of the required dosage or more.

The primary outcomes included mortality rate and the number of hospitalizations due to AD. The number of AD admissions was calculated after the index date. The secondary outcomes included sepsis, cerebrovascular or cardiovascular event, respiratory failure, initiation of long-term dialysis, admission related to viral infection (viral upper respiratory tract infection, viral pneumonia, viral meningitis, viral gastroenteritis), DKA or HHS, acute hepatitis, or newly-onset malignancy. The diagnosis of respiratory failure, long-term dialysis, and malignancy had to be verified by the presence of CIC. The occurrence of a cerebral or cardiovascular event, DKA or HHS, or acute hepatitis was determined through an emergency room’s principal diagnosis or hospitalization. Each patient was followed until the date of event occurrence, death, or 31 December 2013, whichever came first.

### Statistical Analysis

We performed propensity score matching in order to compare the metformin and non-metformin groups. The propensity score was calculated by logistic regression model, using metformin as the dependent variable. The independent variables used to construct the logistic regression model are listed in **Table 1**, including demographics (gender and age), types of autoimmune diseases, surrogate variables of disease severity, comorbidities, glucose-lowering medications, immunomodulatory medications, and other medications that may influence the occurrence of outcome, and the index date. We matched a patient in the non-metformin group with two corresponding patients in the metformin group (13). We carried out the matching using the greedy nearest neighbor algorithm with a caliper width of 0.2 times the pooled standard deviation of the propensity score.

**Table 1:** Characteristics of the study patients before and after propensity score matching.

Variable	Before matching				After matching	
	Metformin (*n* = 3,015)	Non-metformin (*n* = 344)	*P*-value	Metformin (*n* = 678)	Non-metformin (*n* = 339)	*P*-value
Characteristic						
Age (years)	59.8 ± 11.6	63.7 ± 12.2	<0.001	63.2 ± 11.1	63.4 ± 12.2	0.733
Age ≥ 65 years	1024 (34.0)	169 (49.1)	<0.001	309 (45.6)	164 (48.4)	0.398
Female gender	2,332 (77.3)	274 (79.7)	0.332	541 (79.8)	270 (79.6)	0.956
Existing autoimmune diseases in enrollment			0.244			0.990
Rheumatoid arthritis	1,882 (62.4)	205 (59.6)		415 (61.2)	202 (59.6)	
Sjogren’s syndrome	232 (7.7)	26 (7.6)		53 (7.8)	26 (7.7)	
Systemic lupus erythematosus	435 (14.4)	61 (17.7)		118 (17.4)	61 (18.0)	
Polymyositis + dermatomyositis	84 (2.8)	5 (1.5)		10 (1.5)	5 (1.5)	
Crohn’s disease + ulcerative colitis	59 (2.0)	4 (1.2)		9 (1.3)	4 (1.2)	
Other	323 (10.7)	43 (12.5)		73 (10.8)	41 (12.1)	
Severity						
Plasmapheresis or plasma exchange	187 (6.2)	38 (11.0)	0.001	77 (11.4)	36 (10.6)	0.724
Admission due to autoimmune disease	1,381 (45.8)	157 (45.6)	0.954	320 (47.2)	153 (45.1)	0.534
DKA or HHS	49 (1.6)	9 (2.6)	0.181	16 (2.4)	9 (2.7)	0.775
Autoimmune diseases duration (year)	7.0 ± 4.5	6.9 ± 4.9	0.949	6.6 ± 4.6	6.9 ± 4.9	0.299
Comorbidity						
Hypertension	1,457 (48.3)	197 (57.3)	0.002	380 (56.0)	192 (56.6)	0.858
Dyslipidemia	628 (20.8)	62 (18.0)	0.222	121 (17.8)	61 (18.0)	0.954
Gout	196 (6.5)	28 (8.1)	0.248	47 (6.9)	28 (8.3)	0.445
Coronary artery disease	364 (12.1)	48 (14.0)	0.314	83 (12.2)	45 (13.3)	0.640
Chronic kidney disease	278 (9.2)	55 (16.0)	<0.001	100 (14.7)	53 (15.6)	0.710
Interstitial lung disease	55 (1.8)	6 (1.7)	0.916	14 (2.1)	6 (1.8)	0.749
Malignancy	165 (5.5)	12 (3.5)	0.119	22 (3.2)	12 (3.5)	0.805
Hepatitis B virus infection	42 (1.4)	7 (2.0)	0.347	15 (2.2)	7 (2.1)	0.879
Hepatitis C virus infection	105 (3.5)	8 (2.3)	0.259	11 (1.6)	8 (2.4)	0.413
Stroke	135 (4.5)	24 (7.0)	0.039	42 (6.2)	23 (6.8)	0.717
Medication						
Hypoglycemic drugs						
Glinide	176 (5.8)	48 (14.0)	<0.001	83 (12.2)	43 (12.7)	0.840
Alpha glucosidase inhibitors	183 (6.1)	55 (16.0)	<0.001	84 (12.4)	50 (14.7)	0.294
Sulfonylurea	1,705 (56.6)	248 (72.1)	<0.001	503 (74.2)	246 (72.6)	0.580
DPP-4 inhibitor	123 (4.1)	18 (5.2)	0.312	31 (4.6)	17 (5.0)	0.754
Thiazolidinediones	107 (3.5)	15 (4.4)	0.446	33 (4.9)	15 (4.4)	0.754
Insulin	287 (9.5)	38 (11.0)	0.364	73 (10.8)	37 (10.9)	0.943
Autoimmune drugs						
Hydroxychloroquine	997 (33.1)	121 (35.2)	0.432	246 (36.3)	118 (34.8)	0.644
Steroid	1,887 (62.6)	241 (70.1)	0.006	485 (71.5)	236 (69.6)	0.526
Cyclosporine	102 (3.4)	10 (2.9)	0.641	18 (2.7)	10 (2.9)	0.786
Cyclophosphamide	66 (2.2)	9 (2.6)	0.611	21 (3.1)	9 (2.7)	0.694
Azathioprine	211 (7.0)	26 (7.6)	0.701	56 (8.3)	26 (7.7)	0.745
Methotrexate	655 (21.7)	73 (21.2)	0.830	156 (23.0)	73 (21.5)	0.596
Sulfasalazine	628 (20.8)	76 (22.1)	0.585	155 (22.9)	75 (22.1)	0.791
Other drugs						
Antiplatelet (Aspirin or Clopidogrel)	611 (20.3)	71 (20.6)	0.870	141 (20.8)	70 (20.6)	0.956
Statin	761 (25.2)	69 (20.1)	0.035	136 (20.1)	69 (20.4)	0.912
NSAID	2,390 (79.3)	269 (78.2)	0.643	545 (80.4)	266 (78.5)	0.473
Cox-2	1,293 (42.9)	151 (43.9)	0.720	309 (45.6)	149 (44.0)	0.624

DKA, diabetic ketoacidosis; HHS, hyperglycemic hyperosmolar state; DM, diabetes mellitus; DPP4, dipeptidyl peptidase 4; NSAID, non-steroidal anti-inflammatory drug.

After propensity score matching, the characteristics of the metformin and non-metformin groups were compared using the t-test for continuous variables and the chi-square test for categorical variables. Mortality was reported as the proportion of events and incidence density (number of events per 100 person-years). We compared the risk of mortality between the two groups with a Cox proportional hazard model and adopted a Poisson regression model to compare the annual number of admissions between the metformin and non-metformin groups. We compared each secondary outcome between the two groups using a sub-distribution hazard model that considered death a competing risk (14). We considered a *p*-value < 0.05 to be statistically significant. Data analysis was conducted using Stata 13 software (StataCorp LLC, College Station, TX, US) and SAS software version 9.4 (SAS Institute, Cary, NC).

## Results

### Eligible Patients

**Figure 1:**
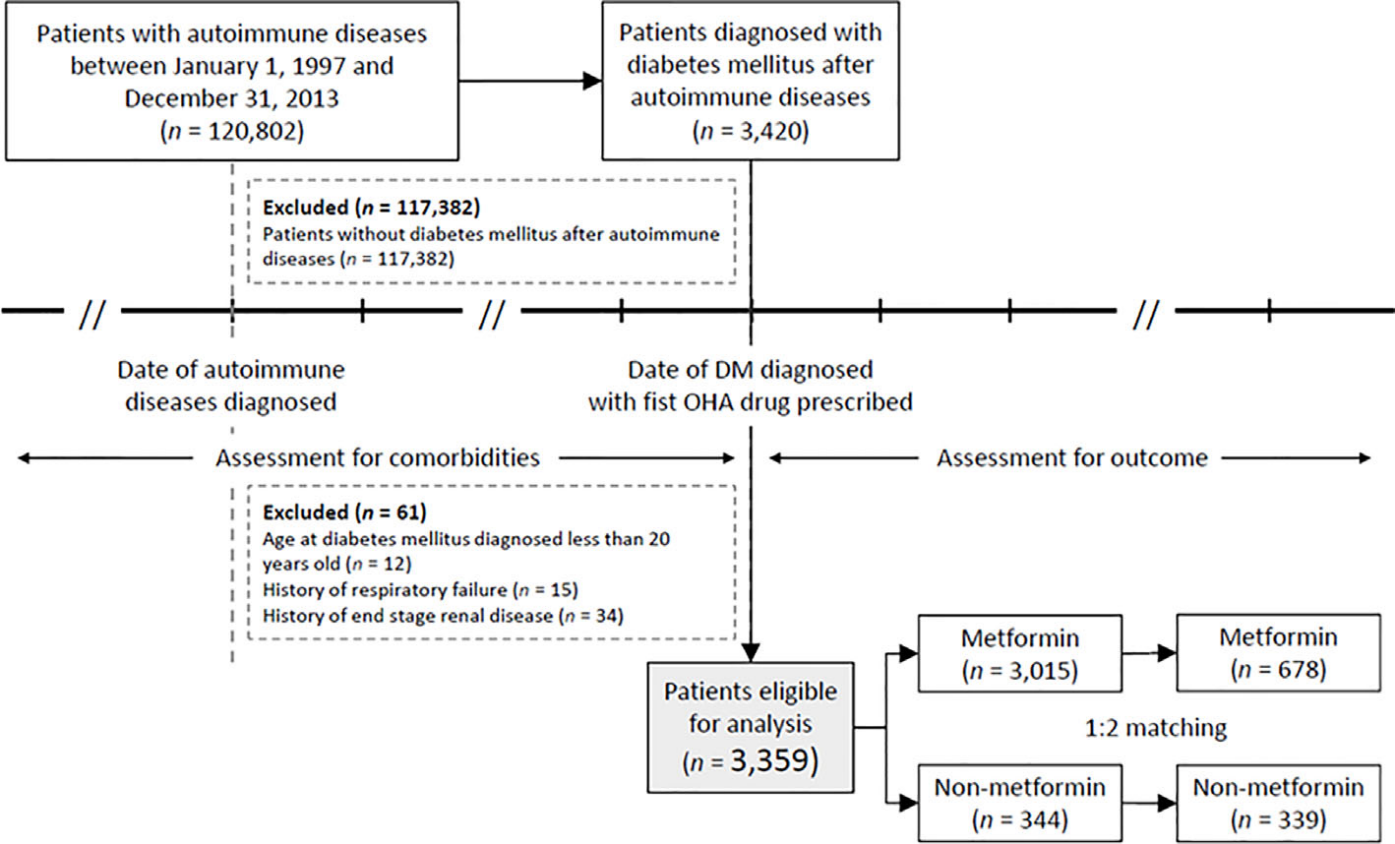
Study design and patient inclusion criteria.

We found a total of 120,802 patients with AD between January 1, 1997 and December 31, 2013. Of those, 117,382 patients were excluded because they did not have a type 2 diabetes mellitus (T2DM) diagnosis after their AD diagnosis; only 3,420 AD patients were diagnosed with T2DM (2.83%). We further excluded patients under the age of 20 years old (n = 12), with a history of respiratory failure (n = 15), or with end-stage renal disease (ESRD, n = 34). Ultimately, 3,359 patients (2.78%) were eligible for analysis. After reviewing their medication history, we observed that 3,015 patients (89.75%) were found to be prescribed metformin, while only 344 patients (10.24%) were prescribed other anti-diabetic agents (**Figure 1**).

### Baseline Characteristics for Study Participants

**Table 1** lists patient characteristics before and after propensity score matching. Prior to matching, the metformin group had 3,015 patients, and the non-metformin group had 344 patients. Patients in the metformin group had a younger age (59.8 ± 11.6 vs. 63.7 ± 12.2, *P* < 0.001) and a similar prevalence rate of underlying autoimmune disease (*P* = 0.244). However, the AD disease activity differed, with patients in the metformin group having fewer episodes of plasmapheresis or plasma exchange and an increased interval between the autoimmune disease and the T2DM diagnosis. Furthermore, patients in the metformin group had a lower prevalence of comorbidities (e.g., hypertension, chronic kidney disease, and stroke), were less likely to be prescribed such medications as glinides, alpha glucosidase inhibitors or sulfonylurea, and steroids, and were more likely to be prescribed a statin (*P* < 0.05).

After propensity score matching, all the parameters shown in **Table 1** were balanced between the metformin and non-metformin groups (all *P* > 0.2). In the end, 678 and 339 patients remained in the metformin group and non-metformin group, respectively, for further analysis.

### Primary Outcomes

**Figure 2:**
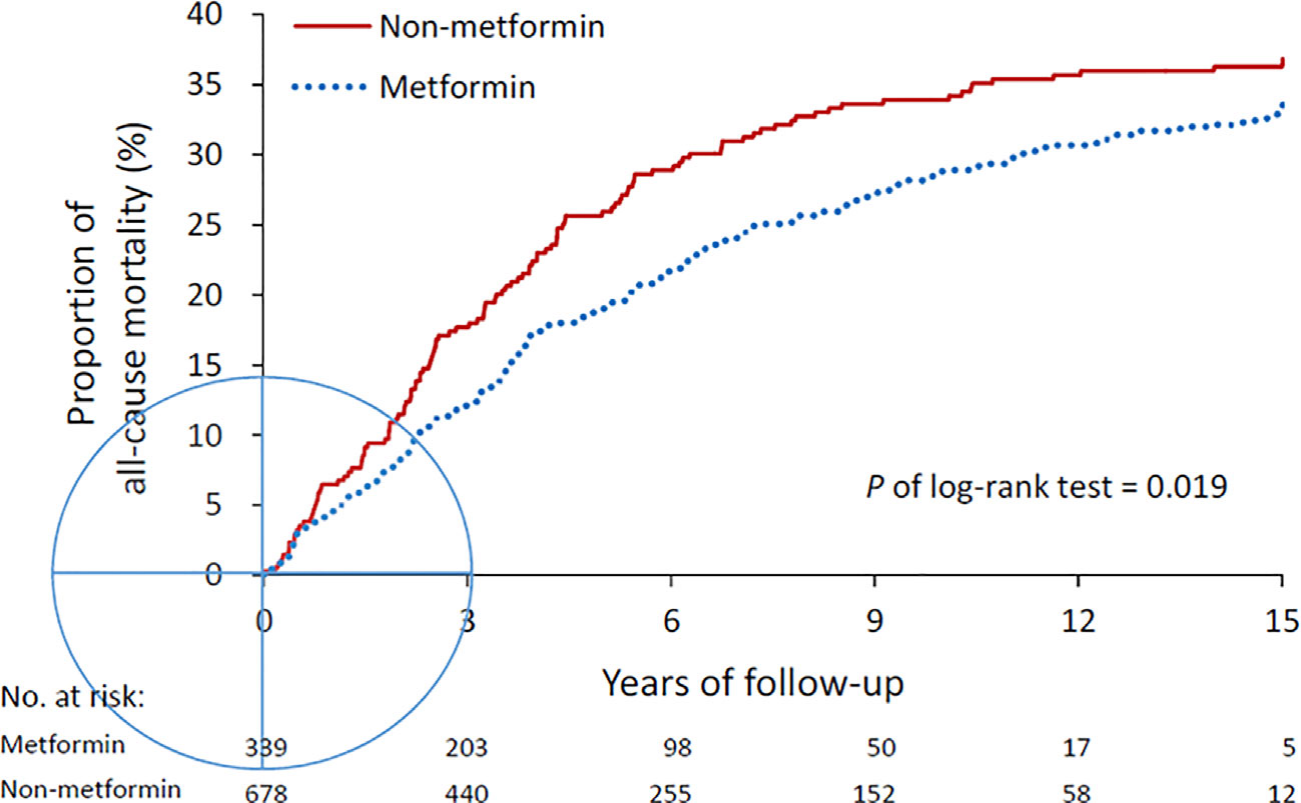
All-cause mortality in the metformin and non-metformin groups. Mortality was compared using log-rank test.

With a mean of 5.2 years (*SD* = 3.8 years) of follow-up duration, the event rate of all-cause mortality was 228 (33.6%) in the metformin group and 125 (36.9%) in the non-metformin group. The risk of all-cause mortality was significantly lower in the metformin group (Hazard ratio [CI], 0.77; 95% confidence interval [CI], 0.62–0.96). The cumulative incidence of mortality during the follow-up period was also significantly lower in metformin users compared to non-metformin users (*P* of log-rank test = 0.019, **Figure 2**). The annual number of admissions for AD was significantly lower in the metformin group (0.35 ± 0.81) than the non-metformin group (0.43 ± 1.08) with a rate ratio of 0.81 (95% CI, 0.73–0.90) (**Table 2**).

**Table 2:** Event numbers and incidence density of the primary outcomes between the study cohorts.

Variable	Metformin(*n* = 678)	Non-metformin(*n* = 339)	*P*-value
All-cause mortality			
Follow-up (years), mean ± SD	5.5 ± 4.1	4.7 ± 3.7	
Event number, n (%)	228 (33.6)	125 (36.9)	
Incidence density (95% CI)^§^	6.15 (5.35–6.95)	7.79 (6.42–9.16)	
Hazard ratio (95% CI)	0.77 (0.62, 0.96)	Reference	0.020
Annual number of admissions for autoimmune disease			
Mean ± SD	0.35 ± 0.81	0.43 ± 1.08	
Rate ratio (95% CI)	0.81 (0.73, 0.90)	Reference	<0.001

SD, standard deviation; CI, confidence interval.

^§^Incidence density (ID), event numbers per 100 person-years.

### Secondary Outcomes

We observed that metformin had a neutral effect on secondary outcomes, except for chronic respiratory failure and long-term dialysis (**Table 3**). The risk of chronic respiratory failure was significantly reduced in the metformin group (HR, 0.56; 95% CI, 0.31–0.99), as was the risk of long-term dialysis (HR, 0.30; 95% CI, 0.14–0.65) (**Table 3**).

**Table 3:** Secondary outcome between metformin and non-metformin groups.

Outcome	Metformin (*n* = 678)	Non-metformin (*n* = 339)	Metformin *vs. *Non-metformin^§^	
			HR (95% CI)	*P*-value
Organ damage				
Vascular outcomes^#^	106 (15.6)	41 (12.1)	1.26 (0.88, 1.80)	0.216
Respiratory failure	25 (3.7)	21 (6.2)	0.56 (0.31, 0.99)	0.048^*^
ESRD on dialysis	11 (1.6)	16 (4.7)	0.30 (0.14, 0.65)	0.002^*^
Acute hepatitis	6 (0.9)	1 (0.3)	2.91 (0.35, 24.11)	0.323
Infectious diseases				
Sepsis	78 (11.5)	42 (12.4)	0.86 (0.60, 1.26)	0.442
Virus infection^¶^	23 (3.4)	13 (3.8)	0.86 (0.44, 1.69)	0.661
Diabetes-related outcome				
DKA or HHS	28 (4.1)	6 (1.8)	2.28 (0.95, 5.51)	0.066
Newly-onset malignancy	52 (7.7)	30 (8.8)	0.81 (0.52, 1.26)	0.350

HR, hazard ratio; CI, confidence interval; DKA, diabetic ketoacidosis; HHS, hyperglycemic hyperosmolar state; ESRD, end-stage renal disease.

^§^estimated using Fine and Gray (1999) sub-distribution hazard model that considered all-cause mortality as a competing risk.

^#^Vascular outcomes include stroke or acute coronary syndrome.

^¶^Virus infection includes viral meningitis, gastroenteritis, flu and herpes infections.

*indicates p < 0.05.

### Subgroup Analysis

**Figure 3:**
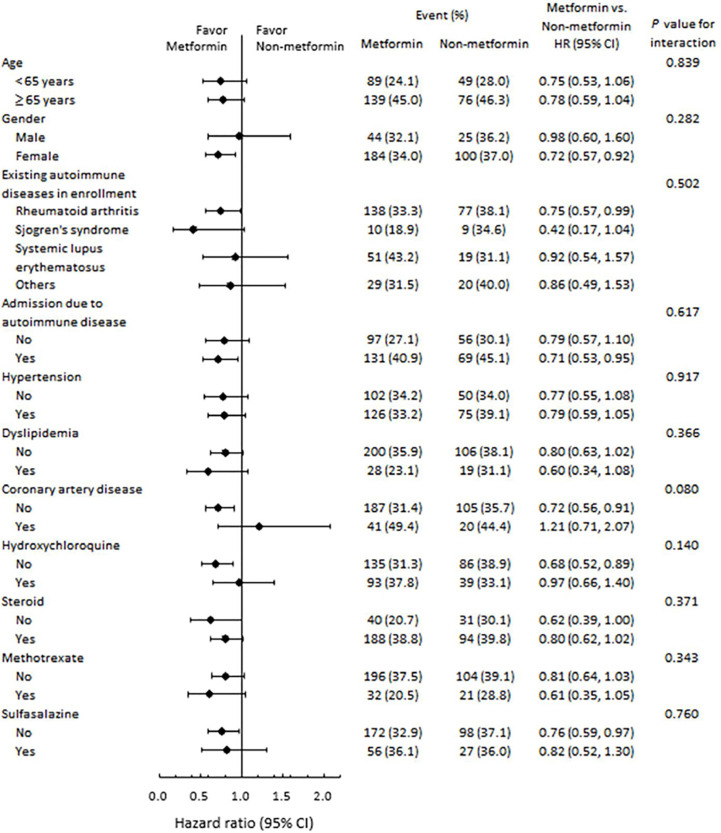
Subgroup analysis of all-cause mortality. The beneficial effects of metformin on risk of mortality were similar across various strata.

**Figure 3** presents the subgroup analysis for all-cause mortality stratified by age, gender, immune diseases, and various medication. The beneficial effects of metformin on mortality were similar across all strata (*P* for interaction > 0.05).

## Discussion

This large AD cohort study demonstrates that patients prescribed metformin have been associated with a significantly decreased rate of all-cause mortality and annual admissions compared to those who do not take metformin but still have a similar incidence rate of sepsis death, vascular event, viral infection, diabetic ketoacidosis, acute hepatitis, or new-onset malignancy. Whether exposure to metformin has a deteriorating or neutral effect on kidney function is still a question of debate (15), but in this study, we prove that metformin may have benefits related to reducing end-stage renal disease and respiratory failure in diabetic AD patients. While some studies have been performed on metformin and reducing mortality in cardiovascular events, hepatoma, heart failure, and atherothrombosis (16), no specific research dealing with the metformin effect on AD has yet been published.

In the current study, the clinical vascular outcome comparison between metformin users and non-metformin users seems to be neutral, which is not in line with previous studies on the protective effect of metformin with regard to the mortality of cardiovascular events, heart failure, and atherothrombosis (16). This discrepancy may be attributed to the underlying more pronounced inflammation of AD patients, which may offset the potential beneficial effect of metformin in this cohort (17, 18). Therefore, the detailed mechanism of metformin regarding cardiovascular events in AD patients warrants additional study.

Chronic renal disease patients account for 12% to 14% of all AD patients, including those with lupus glomerulonephritis (19, 20), vasculitis (21), thromboembolism (22), scleroderma renal crisis (23), interstitial nephritis (24), and drug toxicity (25). Most AD-related chronic kidney diseases are associated with inflammation and oxidative stress (26). Previous studies have shown that diabetic nephropathy, with an incidence rate of around 4%~6% in Asian populations, may be related to oxidative stress (27). Our study results demonstrated that the risk of ESRD is significantly lower in the metformin group. This observation may be explained by the anti-inflammatory effects of metformin.

Likewise, respiratory failure can be lethal in AD (28). The relationship between respiratory failure and T2DM has been discussed before (29, 30). Previous studies have revealed that patients with cystic fibrosis and T2DM had worse outcomes compared to those without T2DM (31), in which oxidative stress was a potential deteriorating marker (32, 33). Our large cohort study indicated that metformin may significantly reduce the respiratory failure rate in AD patients with T2DM, which further shows its extra benefit in immune modulation.

Our study has some limitations that should be mentioned at this point. First, the exact percentage of AD in our cohort was unknown since we could only establish target AD patients through a mixture of different ICD-9-CM codes representing different AD, which could potentially result in selection bias. For example, antiphospholipid syndrome is an autoimmune disorder that may manifest vascular events and often appears in AD patients, but it has no specific ICD-9-CM code. To overcome that problem, we collected some of the medications that may be used to treat antiphospholipid syndrome, such as aspirin and warfarin, and matched them when possible. Second, the enrolled patients were mostly Taiwanese, so the external validity of these results may be questionable. Additional studies for other ethnic groups are required to verify our results. However, our large-scale study may provide an insight for long-term treatment of T2DM in patients with AD.

## Conclusion

In actual usage, metformin seems to have benefits beyond T2DM control with regard to reducing real-world all-cause mortality and admission rates, as well as minimizing end-organ injury in lungs and kidneys among autoimmune disease patients.

## Data Availability Statement

The datasets used and/or analyzed during the current study are available from the corresponding author on reasonable request.

## Ethics Statement

The studies involving human participants were reviewed and approved by National Cheng Kung University Hospital. Written informed consent for participation was not required for this study in accordance with the national legislation and the institutional requirements.

## Author Contributions

All authors contributed to the acquisition of research data. C-YL and Y-JS conducted the literature review, data analysis, and drafted the manuscript. C-YL and Y-JS are the guarantors of this work, have full access to all the data in the study, and take responsibility for the integrity of the data and the accuracy of the data analysis. All authors contributed to the article and approved the submitted version.

## Funding

This study was supported by research grants from the National Cheng Kung University Hospital (NCKUH-10903023 and NCKUH-10801002), the Ministry of Science and Technology (MOST 108-2314-B-006-007-MY2), and Chang Gung Memorial Hospital [grant number CORPG8K0131, NMRPG8G6183].

## Conflict of Interest

The authors declare that the research was conducted in the absence of any commercial or financial relationships that could be construed as a potential conflict of interest.
