# Supplementary material for: Reduced Mortality Associated With the Use of Metformin Among Patients With Autoimmune Diseases
Source: Front Endocrinol (Lausanne). 2021 Apr 23;12:641635. doi: 10.3389/fendo.2021.641635 (PMC8104028; doi:10.3389/fendo.2021.641635)
Supplement: Supplementary file 1 [file Table_1.docx]

| Ankylosing spondylitis | 7200 |
| --- | --- |
| Autoimmune diseases | 430*~437* |
| Behcet’s disease | 136.1 |
| Cancer | 140*~208* |
| Coronary artery disease | 411.1 |
| Diabetes | 250* |
| Diabetes Ketoacidosis or hyperosmolar hyperglycemic state | 250.1*, 250.2*, 250.3* |
| Dyslipidemia | 272* |
| End stage kidney disease | 585* |
| Gout | 274* |
| Hepatitis | 277.4, 570, 572.8, 573.3, 573.8, 576.8, 782.4 |
| Hypertension | 401*, 402*, 403*, 404*, 405* |
| Inflammatory bowel disease | 555, 556 |
| Psoriatic disease | 6960, 6961 |
| Rheumatoid arthritis | 7140 |
| Sjogren's syndrome | 7102 |
| Systemic lupus erythematosus | 7100 |
| Vasculitis | 446.0, 446.2, 446.4, 446.5, 443.1, 446.7, |

Supplementary Table 1.

The International Classification of Diseases, Ninth Revision, Clinical Modification (ICD-9-CM) codes used in this study.
